# Anti-resorptive agents in neuroendocrine bone metastases – A systematic review

**DOI:** 10.1016/j.jbo.2026.100789

**Published:** 2026-07-24

**Authors:** Marianne S. Elston, Colin Chan Chui, Jade A.U. Tamatea, Ben Lawrence, Veronica Boyle

**Affiliations:** a Endocrinology Unit, Te Whatu Ora Waikato, Waikato Hospital, New Zealand; b Waikato Clinical Campus, University of Auckland, Hamilton, New Zealand; c Department of Medical Oncology, Te Whatu Ora Te Toka Tumai Auckland, New Zealand; d Department of Oncology, University of Auckland Waipapa Taumata Rau, New Zealand

**Keywords:** Neuroendocrine neoplasm, Neuroendocrine tumour, Bisphosphonates, Diphosphonates, Denosumab, Bone metastases

## Abstract

**Purpose:**

Bone metastases are common in patients with metastatic neuroendocrine neoplasms (NENs) and frequently result in skeletal complications. The current role of anti-resorptive agents for these patients is not known.

**Methods:**

A systematic review process was conducted according to the Preferred Reporting Items for Systematic Reviews and Meta-Analyses (PRISMA) guidelines and a PICOS framework searching the Embase, Medline, PubMed, Google Scholar, Cochrane library and Web of Science databases to identify studies reporting the use of anti-resorptive agents in patients with well-differentiated NENs who have bone metastases.

**Results:**

A total of 2424 articles were identified with 104 selected for full text review. After review, five retrospective studies were included in the review. Study design was heterogenous, and treatment details typically poorly described. A total of 348 patients were included in the review, of whom 206 received anti-resorptive treatment. One study (*N* = 19) reported an increase in overall survival with use of monthly bisphosphonates whereas no difference in survival with use of anti-resorptive agents were identified in three studies. The response to anti-resorptive therapy was positively associated with overall survival in one study. One study reported a significant reduction in SRE in the group receiving anti-resorptive therapy. Significant toxicity from anti-resorptive therapy was reported in 15.2% of patients in one study whereas treatment complications were not documented in the other three studies.

**Conclusion:**

Currently, there are insufficient data as to the role of anti-resorptive agents in patients with NENs and a prospective trial is needed.

## Highlights

• Neuroendocrine neoplasm bone metastases frequently cause skeletal complications.
• Anti-resorptive therapy reduces skeletal-related events for many other cancer types.
• Only retrospective data are available for neuroendocrine neoplasms.
• The role of anti-resorptives in neuroendocrine neoplasms is uncertain.

## Graphical abstract

**Figure:**
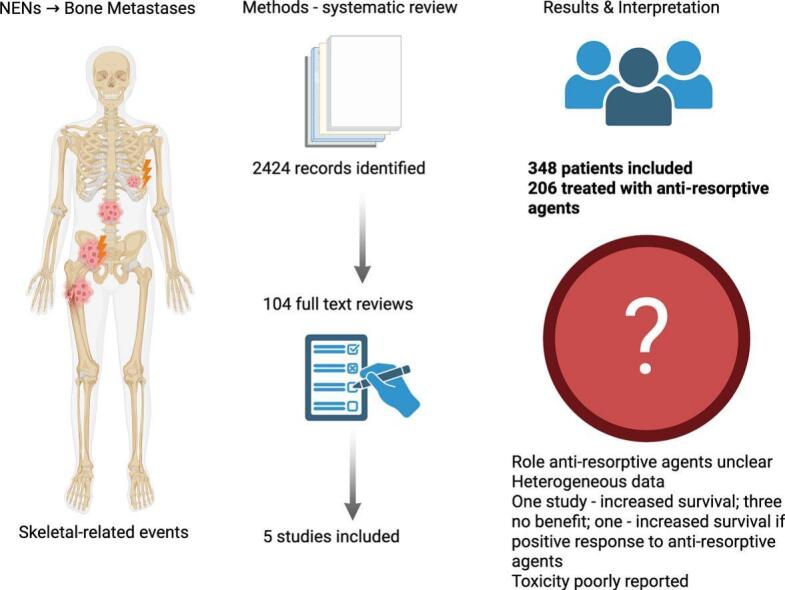

## 1 Introduction

Neuroendocrine neoplasms (NENs) are a heterogenous group of tumours of varying aggressiveness arising from the neuroendocrine cells within multiple tissue types, most commonly gastrointestinal tract and lung [1]. Whilst originally considered rare, the diagnosis of NENs is increasing and given often-long survival, are more prevalent than previously thought. In around half, metastases are present at diagnosis, typically liver and/or regional lymph nodes [1], [2]. Bone metastases, occur in around 18% of patients with NENs, and are predominantly osteoblastic, often multifocal and commonly affect the axial skeleton [3]. When allowing for frequency of primary site, lung primary appears to be associated with a higher proportion developing metastases as compared with pancreas or small intestine primary [4]. Data is conflicting as to whether bone metastases are more likely to occur in those with higher grade tumours [3], [4], [5] and interpretation is difficult due to the various imaging modalities used in studies with 68Ga-DOTATOC PET/CT having a higher sensitivity for detection of bone metastases than anatomical imaging such as CT [6]. Bone metastases are commonly symptomatic and may be associated with pain, and skeletal-related events (SREs) – i.e. pathological fracture, spinal cord compression, or intervention by surgery or radiotherapy. SREs occur in around a quarter of patients with bone metastases [3], [7], [8].

The presence of bone metastases in patients with NENs has been associated with reduced survival as compared to those with metastatic disease but without bone metastases (48.8 months vs 87.4 months) [3]. Current standard systemic treatment options for patients who have metastatic disease from NENs include somatostatin analogues, chemotherapy and peptide receptor radionuclide therapy (PRRT) with limited evidence to specifically address the response of bone metastases.

Use of anti-resorptive therapies (ART), such as bisphosphonates and the RANK Ligand inhibitor, denosumab have been demonstrated to reduce the rate of SREs in a variety of tumour types including breast, prostate, myeloma, renal cell carcinoma, non-small cell lung cancer [9], [10], [11], [12], [13], [14]. In patients with NENs, the response to therapy that could have activity in bone (bisphosphonates, denosumab, local radiotherapy) has been reported to independently predict mortality [15], but management is based on little data with a lack of prospective trials. Current NEN guidelines typically either do not mention use of pharmacological bone-modifying therapies or give little guidance as to agent and/or dosing regimen.

The objective of this review was to summarise the best evidence for the use of ARTs for bone metastases in patients with well-differentiated NENs.

## 2 Methods

The systematic review was registered in the PROSPERO database (CRD42024534738), and the review process was conducted according to the Preferred Reporting Items for Systematic Reviews and Meta-Analyses (PRISMA) guidelines [16]. A PICOS framework was used (Table 1). PubMed, Medline, EMBASE, Web of Science, Cochrane Library and the first 200 results from Google Scholar [17] electronic databases were searched from database inception to the 29th of April 2024. Search strategies are detailed in Supplementary Table S1. No date or language restrictions were used for the initial search. Search results were exported to Mendeley Reference Manager (Version 2.128.0) and duplicates were removed. The search results were initially screened independently by title and abstract by three authors (MSE, CCC, VB). The decision to include a study for full text review was based on the consensus of at least two reviewers. The third reviewer would mediate if consensus was not met. This process was repeated for the review of full text articles. Studies conducted in animals and of patients with only poorly differentiated neuroendocrine neoplasms were excluded as were patients with pheochromocytoma/ paraganglioma and Merkel cell carcinomas. Additionally, studies reporting the use of bisphosphonate for the treatment of hypercalcemia in patients with neuroendocrine neoplasms were excluded.

**Table 1:** PICOS approach to the research question.

P	Population – people with neuroendocrine neoplasms with bone metastases
I	Intervention – anti-resorptive agent – bisphosphonates (all) or denosumab (dose, interval)
C	Comparison intervention – no intervention
O	Outcome measures • Survival • Skeletal-related event-free survival • Skeletal-related events (pain, fractures, cord compression, surgical intervention to bone) • Treatment complications (hypocalcaemia, osteonecrosis of the jaw, atypical femoral fracture)
S	Study designs – all

Following study selection, data were extracted into Excel using data tables organised by study type. Key datapoints for extraction included study design and participant demographic information, primary neuroendocrine neoplasm, tumour grade, skeletal events and complications, treatment given, treatment outcomes, and survival.

The quality of each study was assessed using the JBI critical appraisal tool [18] and assessed independently by three authors (MSE, CCC and VB). Where relevant information was missing, corresponding authors of the respective publications were contacted to obtain additional information.

## 3 Results

**Fig. 1:**
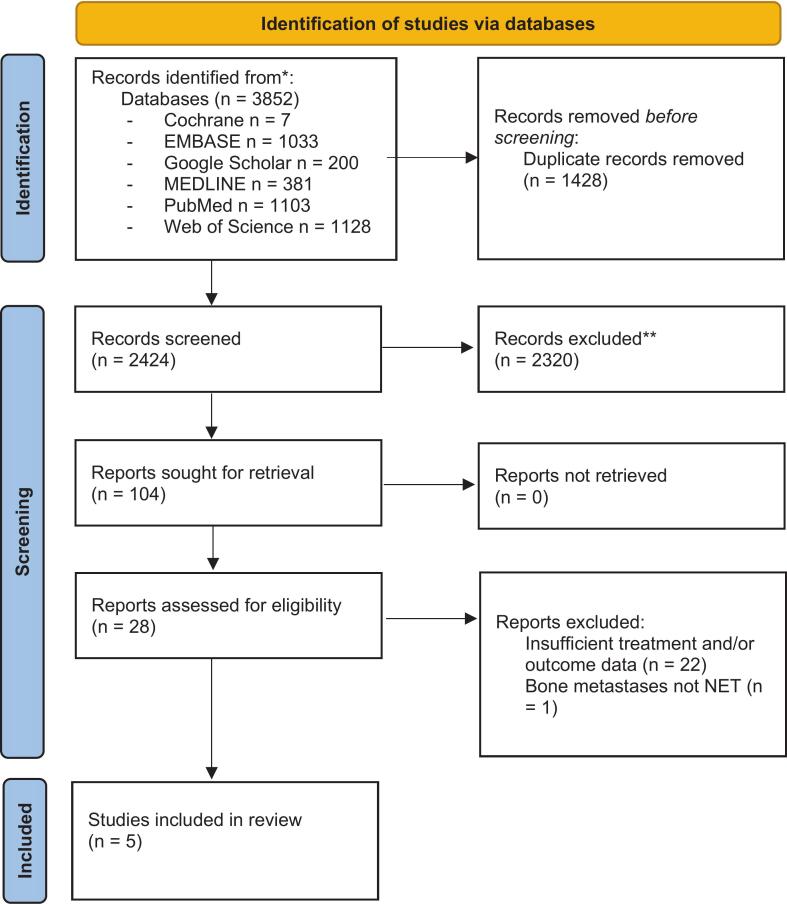
PRISMA flow diagram.

A total of 3852 records were identified from the initial search (Fig. 1). Following title and abstract screening, 104 articles were retrieved for full-text review and of these 28 were considered for inclusion. Most of the articles identified for full text review were excluded after utilising the critical appraisal tool due to insufficient information. No prospective studies were identified. Five retrospective studies comprising four full text articles and one poster were included in the final review [2], [5], [15], [19], [20]. Due to the heterogeneity of the studies (Table 2), a meta-analysis could not be performed and instead a narrative review was performed.

**Table 2:** Included publications.

First author [ref]	Year	Centre	N	Treatment	Clinical details	Outcome
Apostolidis^a^[19]	2017	Single centre (Heidelberg, Germany)	108	Anti-resorptive therapy (43%) - Bisphosphonate^c^ 23 - Denosumab 18 - Both 5 No ART 62 (57%) Radiotherapy 25 (23%) Surgery to bone metastases 0	Median age 59 years Lytic lesions: 29, non-lytic 79 Primary: pancreas 26, small intestine 20, stomach 2, colon/rectum 10, lung/thymus 8, prostate 9, other 8, unknown 25) G1/2–64 (59%) G3–44 (41%) Median Ki-67 12% (range 1–95%)	Pain – not reported SRE – fractures 4 (3.7%), spinal cord compression 2 (1.9%), radiotherapy 25 (23.1%), surgery 0 28.7% experienced at least one SRE No difference in survival, (*p* = 0.25) Median time to SRE 63.8 months with anti-resorptive vs 127 months without, *p* = 0.18 Toxicity - significant toxicity from anti-resorptive in 7/46 (15.2%) (increased creatinine 4 bone pain 2, infusion reaction 1, osteonecrosis of jaw 2)
Scharf [2]	2018	Single centre (Marburg, Germany)	85	Anti-resorptive treatment (72%) - Bisphosphonates^d^ 55 - Denosumab 6 No ART 24 (28%) Radiotherapy 22 (26%) Surgery to bone metastases 3 (3.5%)	Median age 57.3 years Type of metastases – not reported Primary: small intestine 32, pancreas 26, rectum 7, lung 7, duodenum 1, appendix 1, unknown 11 Liver metastases present 80 G1/2–71 (84%) G3 or poorly differentiated – 14 (16%) Median Ki-67 5% (range 1–90)	Pain reported in 36 (42.4%) SRE – fractures 10 (11.8%), spinal cord compression 4 (4.7%), radiotherapy 22 (25.9%), surgery to bone 3 (3.5%) Median time to SRE – not reported OS worse with bone metastases than without, 49 vs 100.8 months, *p* = 0.01 Median OS in patients without symptoms longer than those with symptomatic bone metastases (79.0 vs. 42.0 months; *p* = 0.03). Bone-specific treatment overall survival 49 vs 37.4 months without, *p* = 0.39 Toxicity – not reported
Alexandraki [15]	2019	Multicentre (Greece, Israel, UK)	74	Anti-resorptive treatment 54 (73%) - Bisphosphonates^d^ 41 - Denosumab 13 No ART 20 (27%) Radiotherapy 13 (23%) Surgery to bone metastases not reported	Median age 62 years Type of metastases – not reported Primary: pancreas 22, small intestine 20, lung 11, unknown 12, large bowel 6, thymus 2, breast 1 G1/2–49 (66%) G3–5 (7%) Not stated - 20 Median Ki-67 5% (4–60)	Pain – 19 (25.7%) SRE – not clearly reported Stronger predictor of OS was the outcome of bone-related therapy (HR: 4.753; 95% CI: 1.589–14.213) No difference was seen in the subgroup receiving monthly bisphosphonate treatment and the subgroup receiving other bisphosphonate schemes Toxicity – not reported
Leng [20]	2021	Single centre (China)	19^b^	Anti-resorptive treatment 9 (47%) - Bisphosphonates^e^ 9 (monthly injection) No ART 10 (53%) Radiotherapy 9 (47%) Surgery to bone metastases 19 (100%)	Median age 53 years Type of metastases – not reported Primary: lung 7, GIP 4, thymus 3, unknown 2, bladder 1, uterus 1, breast 1 G1/2–11 G3–8 Median Ki-67 not reported	Pain – 16/19 (84%) SRE – fractures 3 (15.8%), neurological deficit^f^ 18 (94.7%), radiotherapy 9 (47%), surgery for spinal metastases 19 (100%) Univariate analysis – monthly injection bisphosphonate associated with increased OS 15.4 vs 39.8 months, *p* = 0.023 Toxicity – not reported
Mathew [16]	2023	Single centre (Germany)	62	Antiresorptive treatment^g^ 36 - Bisphosphonate^e^ - Denosumab No ART 26 Radiotherapy Surgery to bone metastases	Median age 57 years Type of metastases: unknown 30, osteoblastic 23/32, osteolytic 5/32, mixed 4/32 Primary: pancreas 62 G1–10 (16%) G2–39 (63%) G3–9 (15%) Unknown 4 (7%)	SRE 11/62 (18%) - SRE in 2/36 receiving ART - SRE in 9/25 not receiving ART, *p* = 0.0054 OS – 41 months if SRE vs 67 months without SRE, *p* = 0.185; survival for ART or no ART – not reported Toxicity – not reported

G = World Health Organization tumour grade; ART = anti-resorptive therapy; GIP = gastrointestinal and pancreatic; OS = overall survival; ^a^Abstract and poster only; ^b^All had surgery for symptomatic spinal metastases; ^c^Bisphosphonate type, dose & interval not defined; ^d^Variable bisphosphonate type, dose, and interval including alendronate, zoledronic acid (monthly in 19); ^e^Bisphosphonate not further defined; ^f^Includes all neurology from radiculopathy to paralysis; ^g^Breakdown of those receiving bisphosphonate or denosumab not reported.

*Anti-resorptive treatment (ART) regimens.*

From across the five studies, a total of 348 patients were included, of which 206 received ART (bisphosphonates in 133, denosumab in 42, including 5 patients who received both, unknown in 36). The bisphosphonate type, dose and interval were poorly described. In one study the bisphosphonate administered ranged from a monthly to an annual infusion of 4-5 mg of zoledronic acid, pamidronate infusion or once weekly oral alendronate [15]. The denosumab dosing and interval were not reported in the four studies in which this agent was used [2], [5], [15], [19].

*Skeletal related events.*

Only two studies reported on SRE in relation to ART. A study limited to pancreatic NENs reported a lower rate of SREs in patients who received ART as compared to those who did not receive these agents (2/36 vs 9/26, *p* = 0.0054); time to SRE and overall survival were not reported [5]. The second study reported a shorter median time to SRE in those who received ART compared to those who did not (63.8 months vs 127 months, respectively *p* = 0.1751) [19]. While the other studies did describe SREs in their cohort, they did not report on this in relation to ART.

*Survival.*

One study of NEN patients who had received surgery to spinal metastases (*N* = 19) reported an increased overall survival with use of monthly bisphosphonates (15.4 months in those who did not receive bisphosphonates vs 39.8 months in those that did *p* = 0.023) [20] whereas no difference in survival with use of anti-resorptive agents were identified in the other three studies that reported this (Table 2). In one study, the response to bone-related therapy was associated with overall survival with no difference in outcome with use of monthly bisphosphonates as compared to different anti-resorptive regimens [15]. However, in this study bone-related therapy referred to bisphosphonates, denosumab, external beam radiotherapy or peptide receptor radionuclide therapy rather than ARTs alone [15]. Treatment toxicity was only reported in one study with 15.2% of patients who received bisphosphonates reported as experiencing significant toxicity (two cases each of osteonecrosis of the jaw and bone pain, infusion reaction in one and an increase in creatinine in four [19].

## 4 Discussion

Bone metastases in patients with NENs are frequently symptomatic (46.4%) with 26.2% of patients experiencing either fracture or spinal cord compression with a mean time to SRE of 9.9 months [2]. For patients with stage IV disease, the presence of bone metastases is associated with reduced survival compared to patients without bone metastases [5], [8]. As such, there is a need for treatment options for this group of patients. We did not identify any prospective randomised studies assessing the role of ART in patients with NENs who have bone metastases. Most of the retrospective reports identified contained insufficient outcome data for inclusion in this review.

Of the five studies included, heterogeneity of design, particularly of ART, has meant that a combined analysis cannot be performed. Of the studies which reported treatment with a bisphosphonate, dose and interval varied considerably or were not clearly described. Outcome comparisons between those who received anti-resorptive agents, compared to those who did not was also lacking. Importantly, there was a lack of information on treatment toxicity with only one study reporting toxicity (“significant toxicity” in 15.2% [19]).

Of interest, in the poster by Apostolidis et al., those who received an anti-resorptive agent had a non-significant shorter time to the first SRE than those who did not receive these agents [19]. The reason for this is unclear but as this was a retrospective study, potentially could be due to patients with progressive or symptomatic disease being more likely to be treated with anti-resorptive agents. This contrasts with the study by Mathew et al. which demonstrated a reduction in SREs in patients with pancreatic NENs receiving ART [5]. Leng also demonstrated a benefit with an increase in overall survival was seen in those who received monthly bisphosphonate injections [20]. However, the latter study likely included a different patient population with all patients having had surgery for symptomatic spinal metastases, was not randomised, and only a univariate analysis was performed so factors such as tumour grade were not controlled for, in the small sample. As such, survival differences were likely unrelated to the ART.

Use of ART in other solid tumours can delay or reduce SREs [9], [10], [11], [12], [13], [14]. However, it is unclear as to whether this can be extrapolated to patients with well-differentiated NENs who have bone metastases. Based on the systematic review by Garcia-Torralba [3], most bone metastases in patients with NENs are osteoblastic. This contrasts with most of the large randomised controlled trials of ARTs in some other common tumour types, such as breast cancer, where bone metastases are more likely to be osteolytic or mixed [10], [11], [12], [13]. The mechanism for bone metastases in osteolytic metastases appears to be osteoclast-mediated with important roles of PTHrP and RANK ligand whereas for osteoblastic metastases the mechanism appears to be less well understood but includes increased production of factors simulating osteoblasts such as endothelin-1, BMPs, Wnt agonists, IGF1 and TGF-beta and inhibiting osteoclasts such as ET-1 and OPG (reviewed in [21]). As such, the treatment response of osteoblastic bone metastases to ARTs may differ from that of osteolytic or mixed metastases.

Patients with prostate cancer typically have osteoblastic metastases and have also been demonstrated to show a reduction and longer time to first SREs with zoledronic acid [9]. Use of denosumab has also been demonstrated to delay bone metastases developing in men with prostate cancer [22]. As noted above, patients with NENs also typically have osteoblastic metastases but there are clinically relevant differences in the NEN and prostate cancer populations, and those with prostate cancer may have risk factors for SREs related to androgen-deprivation therapy not usually relevant to patients with NENs. Patients with NENs, however, may also have factors relevant to SRE risk such as excess hormonal secretion (serotonin and/or metabolites, PTHrP, associated Cushing's syndrome etc.) increasing the risk of reduced bone density or altering the risk of treatment complications such as small bowel resection resulting in reduced nutrient absorption and therefore potentially an increased risk of treatment-associated hypocalcemia. Thus, the efficacy and side effect profile of anti-resorptive agents in prostate cancer cannot be extrapolated to NENs.

When compared to zoledronic acid, denosumab may have greater efficacy at delaying skeletal events [23] but is more expensive and a survival advantage has not been demonstrated in other tumour types [24]. Mathew et al. reported no difference between those who received a bisphosphonate as compared to denosumab but the numbers receiving each agent and treatment regimen were not given [5]. Data is conflicting on which agent is associated with a higher risk of complications, such as osteonecrosis of the jaw [25], [26], [27]. This is an important consideration for patients with NENs given the potential for longer survival compared to patients with other tumour types [28].

Strengths and limitations: A strength of this review is the focus on use of ART in well-differentiated NENs, an area which has received relatively little focus to date, despite the prevalence and morbidity associated with bone metastases in these patients. A limitation of this work is the lack of prospective studies in this area. Data quality is a major limitation including the inclusion of data from non-peer reviewed work, the heterogeneity of design and variable amount of information reported, including important NEN classifications such as tumour grade and primary site in patients receiving ART versus those who didn't. As such, clear recommendations are unable to be made as to the benefit or not of ART in this patient population. In the absence of prospective data, if we extrapolate from other tumour types with osteoblastic metastases and allowing for the relatively long survival of patients with NENs we would currently suggest consideration of 12-weekly zoledronic acid infusions as per the protocol by Himelstein et al. [28].

Conclusion: Bone metastases are common in patients with NENs, are frequently symptomatic, and have been associated with reduced survival, but currently there is insufficient data as to the role of anti-resorptive agents in these patients and a prospective trial in this area is needed.

## CRediT authorship contribution statement

**Marianne S. Elston:** Writing – review & editing, Writing – original draft, Supervision, Project administration, Methodology, Investigation, Funding acquisition, Conceptualization. **Colin Chan Chui:** Writing – review & editing, Writing – original draft, Investigation. **Jade A.U. Tamatea:** Writing – review & editing, Supervision, Methodology, Conceptualization. **Ben Lawrence:** Writing – review & editing, Visualization. **Veronica Boyle:** Writing – review & editing, Supervision, Methodology, Investigation, Conceptualization.

## Funding

This work was supported by a New Zealand 10.13039/100005622Health Research Council Health Delivery Activation Grant (23–905,2023).

## Declaration of competing interest

The authors declare the following financial interests/personal relationships which may be considered as potential competing interests: Marianne Elston reports financial support was provided by Health Research Council of New Zealand. If there are other authors, they declare that they have no known competing financial interests or personal relationships that could have appeared to influence the work reported in this paper.
