# Supplementary material for: Anti-resorptive agents in neuroendocrine bone metastases – A systematic review
Source: J Bone Oncol. 2026 Jul 24;60:100789. doi: 10.1016/j.jbo.2026.100789 (PMC13452160; doi:10.1016/j.jbo.2026.100789)
Supplement: Supplementary file 1 [file mmc1.docx]

**Supplementary Table 1.** Search Strategies

**PubMed:** (neuroendocrine OR carcinoid OR "neuro endocrine" OR neuro-endocrine OR argentaffinoma OR gastrinoma OR insulinoma OR glucagonoma OR somatostatinoma OR VIPoma) AND (bisphosphon* OR diphosphon* OR zoledron* OR pamidron* OR clodron* OR ibandron* OR denosumab OR bone-related OR bone-directed OR bone metastases)

**Medline:** (neuroendocrine OR carcinoid OR argentaffinoma OR gastrinoma OR insulinoma OR glucagonoma OR somatostatinoma OR VIPoma) AND (bisphosphon* OR zolendron* OR pamidronate OR clodronate OR ibandronate OR denosumab OR disphosphon* OR bone metastases)

**EMBASE**: (neuroendocrine OR carcinoid OR argentaffinoma OR gastrinoma OR insulinoma OR glucagonoma OR somatostatinoma OR VIPoma) AND (bisphosphon* OR zolendron* OR pamidronate OR clodronate OR ibandronate OR denosumab OR disphosphon* OR bone metastases)

**Web of Science:** (neuroendocrine OR carcinoid OR insulinoma OR glucagonoma OR somatostatinoma OR VIPoma) AND (bisphosphon* OR zoledron* OR pamidronate OR clodronate OR ibandronate OR denosumab OR bone-related OR bone-directed OR bone metastases)

**Cochrane Library:** (neuroendocrine OR carcinoid)

**Google Scholar:** (neuroendocrine OR carcinoid OR “neuro endocrine” OR neuro-endocrine OR argentaffinoma OR insulinoma OR glucagonoma OR somatostatinoma OR VIPoma) AND (bisphosphon* OR diphosphon* OR zoledron* OR pamidronate OR clodronate OR ibandronate OR denosumab OR bone-related OR bone-directed OR bone metastases)
